# In Vitro Investigation Using a New Biomechanical Force–Torque Analysis System: Comparison of Conventional and CAD/CAM-Fixed Orthodontic Retainers

**DOI:** 10.3390/ma17194916

**Published:** 2024-10-08

**Authors:** Francesca Thaden, Linus Hötzel, Hisham Sabbagh, Matthias Mertmann, Andrea Wichelhaus

**Affiliations:** Department of Orthodontics and Dentofacial Orthopedics, LMU University Hospital, LMU Munich, Goethestrasse 70, 80336 Munich, Germany; francesca.thaden@gmail.com (F.T.); linus.hoetzel@med.uni-muenchen.de (L.H.); hisham.sabbagh@med.uni-muenchen.de (H.S.); matthias.mertmann.extern@med.uni-muenchen.de (M.M.)

**Keywords:** biomechanical phenomena, orthodontics, retainer, orthodontic retainer adverse effects, force, torque

## Abstract

(1) Background: After more than a decade since their first description, Inadvertent Tooth Movements (ITMs) remain an adverse effect of orthodontic retainers without a clear etiology. To further investigate the link between ITMs and the mechanical properties of different retainers, the response upon vertical loading was compared in three retainer types (two stainless steel and one nickel–titanium). The influence of different reference teeth was also considered. (2) Methods: Three retainers (R1, R2, R3) were tested in a newly developed biomechanical analysis system (FRANS). They were bonded to 3D-printed models of the lower anterior jaw and vertically displaced up to 0.3 mm. Developing forces and moments were recorded at the center of force. (3) Results: The vertical displacement caused vertical forces (Fz) and labiolingual moments (My) to arise. These were highest in the lateral incisors (up to 2.35 ± 0.59 N and 9.27 ± 5.86 Nmm for R1; 1.69 ± 1.06 N and 7.42 ± 2.65 Nmm for R2; 3.28 ± 1.73 N and 15.91 ± 9.71 Nmm for R3) for all analyzed retainers and with the R3 retainer for all analyzed reference teeth, while the lowest Fz and My values were recorded with the R1 retainer. (4) Conclusions: Displacements of 0.2 mm and larger provided forces and moments which could be sufficient to cause unwanted torque movements, such as ITMs, in all analyzed retainers. Clinicians must be mindful of these risks and perform post-treatment checkups on patients with retainers of all materials.

## 1. Introduction

After teeth have been moved orthodontically, it is necessary to stabilize them in their post-treatment position to prevent relapse [1,2,3]. Orthodontic relapse is not predictable [1] and can lead to unsatisfactory alignment in the long-term post-treatment phase, especially in the lower anterior region, in up to 50% of patients [4]. The treatment result can be stabilized by using fixed or removable retention devices [5]. Fixed bonded retainers have become increasingly popular since their introduction in the 1970s, as they are not dependent upon patient compliance [6,7]. Currently, most orthodontic retainers are made of thin multistranded stainless-steel (SS) wires [8]. Additionally, numerous non-metallic materials can be used for orthodontic retention, such as glass-fiber-reinforced composites [9] and PEEK [10]. In recent decades, computer-aided-design (CAD) and computer-aided manufacturing (CAM) technology have gained importance in all areas of dentistry [11], including orthodontics, where they are being used to manufacture various orthodontic appliances, such as aligners [12]. CAD/CAM has also made it possible to use materials such as superelastic nickel–titanium (NiTi) [13], titanium [14] and Yttria-stabilized zirconia [15] for the manufacturing of orthodontic retainers. Recent randomized controlled trials show that CAD/CAM-fabricated retainers are as effective as conventional SS retainers in maintaining alignment in the short-term [16].

While twisted stainless-steel retainer wires represent the gold standard of orthodontic retention, they have become associated with unexpected complications, known as Inadvertent Tooth Movements (ITMs) [17]. ITMs involve a seemingly intact retainer and can occur as an X-Effect, in which a torque difference develops between two neighboring incisors, or as a Twist-Effect, in which one or both canines develop a labial or a lingual torque [17]. The prevalence of ITMs has been reported to lie between 1.1% and 5% [17,18,19], while some authors claim that moderate cases can be observed in up to 30% of patients [20]. Removal of the affected retainer wire can lead the ITM malocclusion to partly reverse [21] and is the first step in treating ITMs. In severe cases of ITMs, orthodontic retreatment [22,23,24], or even extraction [25], can be required to restore a physiological tooth position and occlusal relationship.

The exact cause of ITMs has yet to be ascertained, although many hypotheses have been advanced. Some authors claim that patient-dependent features, such as pretreatment incisor inclination [18], age at debonding [18,20], the persistence of oral dysfunctions [26], or intercanine distance [27] could be significant for the development of ITMs. Others suggest an iatrogenic influence, concluding that an important role is played by operator experience and wire passivity during bonding [17,24,28]. Still, others propose that the cause of ITMs lies in the retainer wire itself, using “wire syndrome” [29] or “unintentionally active retainers” [8] as synonyms for Inadvertent Tooth Movements. A recent study highlights the role of the bonding site, theorizing that, in a bonding site without proper isolation, ITMs could be the clinical expression of teeth rotating around the retainer wire [30].

There have been several biomechanical studies regarding retainers in the past, most of which have focused predominantly on multistranded stainless steel retainers or on their comparison to other materials such as gold or glass-fiber-reinforced composite [9,31,32,33,34,35]. Since the development of newer materials for orthodontic retainers, several studies have compared stainless-steel and CAD/CAM-manufactured retainers in a clinical setting [36,37,38,39], while only a few publications have analyzed these retainers from a biomechanical standpoint. In 2023, Roser et al. [40] analyzed a conventional SS retainer and six different CAD/CAM-manufactured retainers in respect to aging and maximum load capacity. They found that the SS retainer performed better than all analyzed CAD/CAM-manufactured retainers in both aspects, claiming that SS retainers could still be considered the gold standard.

In contrast to the previously cited biomechanical studies [31,34,35,40], we developed a novel measurement system which was not only specifically designed for the biomechanical testing of retainers but also capable of analyzing the effect of vertical loading on two biomechanically isolated symmetrical reference teeth with the aim of further understanding the phenomenon of ITMs. The present study also aims to provide useful information for clinicians by comparing three different commercially available retainers (two conventionally fabricated SS retainers and one CAD/CAM-manufactured NiTi retainer) in regard to force and moment development upon loading. While the two SS retainers represent the gold standard in fixed orthodontic retention, the CAD/CAM NiTi retainer was included in our study to compare its performance against conventional retainer materials. The effect of loading on different teeth was also analyzed. 

Therefore, the first hypothesis of this study was that there will be no differences in force/moment development between the CAD/CAM-manufactured nickel–titanium retainer and the two stainless-steel retainers. Another hypothesis was that there will be no differences in the force/moment development between different reference teeth.

## 2. Materials and Methods

### 2.1. Measurement Setup and Specimen

**Figure 1:**
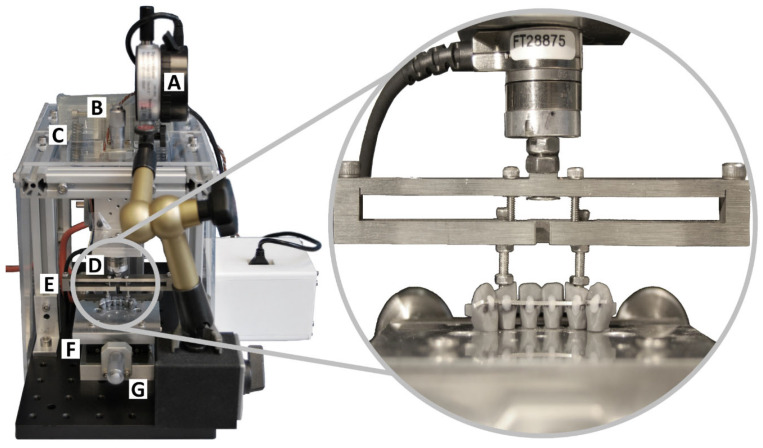
The FRANS setup with all its components. [A] Digital Displacement Measuring Device; [B] micrometer screw gauge and dial gauge for vertical displacement; [C] springs to limit uncontrolled vertical movement; [D] Force/Torque sensor; [E] connector piece for force/moment transmission between sensor and reference teeth; [F] measurement table representing the lower jaw, with placement slots for the experimental setup representing the alveolar sockets; [G] screw gauge for the sagittal displacement of the measuring table. In this instance, the R2 retainer with the lateral incisors as reference teeth is being analyzed.

The Force–Torque Retainer Analysis System (FRANS) was developed in the Biomechanics Laboratory of the Department of Orthodontics and Dentofacial Orthopedics of the Ludwig-Maximilians University Hospital. The FRANS was tested and calibrated with precise weights prior to the experiments. Its purpose was to measure force and torque in relation to a vertical displacement simultaneously in all three spatial planes. Forces and moments were measured at the center of force (CoF), which coincided with the retainer. The measurement setup is shown in Figure 1. It was designed using CAD software (Autodesk Inventor 2021, Autodesk, San Rafael, CA, USA) and had sockets for the placement of the lower canines and incisors (teeth 33, 32, 31, 41, 42, and 43). The sockets were perpendicular to the measurement table, simulating a lower incisor to mandibular plane angle (L1-ML) of 90°.

The table’s sagittal position was able to be altered to ensure a specific angulation of the vertical displacement force to the measured specimen, thus allowing the force to run through the specimen either perpendicularly or at an angle.

The active element of the FRANS was a micrometer screw gauge which, when activated, caused a continuous vertical displacement of a movable element perpendicular to the measurement table. This element was connected by means of coil springs and could only move along the vertical axis, ensuring that no unintentional movement occurred in other spatial planes. The screw gauge was also connected to a dial gauge, which allowed for precise measurement of the obtained displacement with a resolution of 0.01 mm.

The experimental specimens were directly connected to a six-component Force/Torque Sensor (Force resolution 0.0125 N, Moment resolution 0.626 Nmm; Nano17 SI-50-0.5, ATI Industrial Automation, New York, NY, USA) using M3 screws, which were connected to an alterable pair of teeth. This allowed the FRANS to analyze the effects of forces and moments acting upon these two reference teeth as opposed to the whole arch. A computer program was specifically developed within LabVIEW 2020^®^ (NI, Austin, TX, USA) to operate the test stand and to collect and analyze data while the measurements were being performed.

The scan of a patient who had completed orthodontic treatment was used as a template for the lower anterior region, from which the teeth were segmented and isolated using Autodesk Inventor 2021. The used data were taken from the digital archives of the department. These contain anonymized data without patient references so that no informed consent is required for this project according to the European General Data Protection Regulations. A cylindric structure was then added basally to each tooth to simulate the root. The length of the simulated roots was based on average root length values for the respective tooth [41]. For each group, two teeth of the specimen were chosen as reference teeth and were designed with shorter roots and M2 threads along their long axis, thus allowing for the placement of the screws which connect the specimen to the sensor. The shorter roots allowed free movability of the respective teeth within the setup. All teeth in the setup were fabricated using a resin-based additive process with an SLA 3D printer (Formlabs 3Bt, Grey V4 resin, Formlabs GmbH, Berlin, Germany) set to a Z-resolution of 100 µm. The printed objects were postprocessed (washed and cured) following the device manufacturer’s protocol. After this, the inside of the burr holes was threaded manually with an M2 screw tap to accommodate the connecting screws. The teeth were printed en-bloc, ensuring a constant position of the teeth to each other, and separated with a fine cutting disk once the retainer had been bonded. This physical separation allowed each tooth to be biomechanically isolated from its neighboring teeth, with the only connection between them being the retainer.

### 2.2. Measurement Procedure

Three retainer materials (R1, R2, and R3) were compared in this study with respect to their response to vertical loading of up to 0.3 mm (Table 1). Each combination of reference teeth was analyzed (central incisors 31 and 41, lateral incisors 32 and 42, and canines 33 and 43) with a retainer of each type. Groups were formed to compare measurements with the same variables, with n = 5 specimens per group and 45 total specimens. Each specimen was only measured once.

Only measurements which reached 0.3 mm displacement with intact bonding sites were included in final data processing. The number of included measurements can be seen in Table 1. Five measurements from the R1 group were excluded due to bonding failure: two had central incisors, two had lateral incisors, and one had canines as reference teeth. Eight measurements from the R2 group were also excluded due to bonding failure: all five measurements with central incisors as reference teeth were affected, as well as one with lateral incisors and two with canines. One R3 measurement was excluded due to an incomplete failure and had central incisors as reference teeth.

The two stainless steel retainers (R1 and R2) were adapted by using pliers and by hand, respectively. They were prepared with special attention to constant placement height, which was ensured by using a model with a visual marking to bend all retainers. All stainless-steel retainers were prepared by the same operator and checked for wire passivity by an experienced dental technician. The NiTi retainer (R3) was CAD/CAM-manufactured and laser-cut from a superelastic NiTi sheet. Its placement height was determined through CAD software.

All retainers were bonded to the specimen in the middle third of the clinical crown using a silicone transfer jig and flowable composite (Transbond LV, 3M, St. Paul, MN, USA). Then, the connecting screws were placed inside the burr holes parallel to the long axis of the teeth. The specimens were placed into the sockets of the measuring table and the connecting screws were placed into contact with the sensor (magnified detail in Figure 1). The teeth were fixed in their sagittal position using Rendell screws, ensuring force application through the long axis of these teeth. The FRANS was placed inside a temperature-controlled air-chamber, which was set at 36.0 °C ± 1.0 °C to simulate body temperature. The temperature was controlled using a fan heater (PiccoVent, RO/SE Blechverarbeitung GmbH & Co. KG, Bad Birnbach, Germany). It was ensured that the measurement system was calibrated and that all forces and moments were equal to zero before the measurements were commenced.

The measurement was started by activating the micrometer screw gauge. A vertical force was applied continuously at a crosshead speed of 0.5 mm/min ± 0.05 mm/min, extruding the specimen until a displacement of 0.3 mm was reached. During the measurement, the force and torque values were recorded in all three spatial planes at the center of force (CoF). Their values were recorded at a displacement range between 0 and 0.3 mm. In order to prevent collision between the base plate and the measured teeth, the specimen had to be extruded rather than intruded, assuming that the force and torque tensors differ only by sign but not by value.

**Figure 2:**
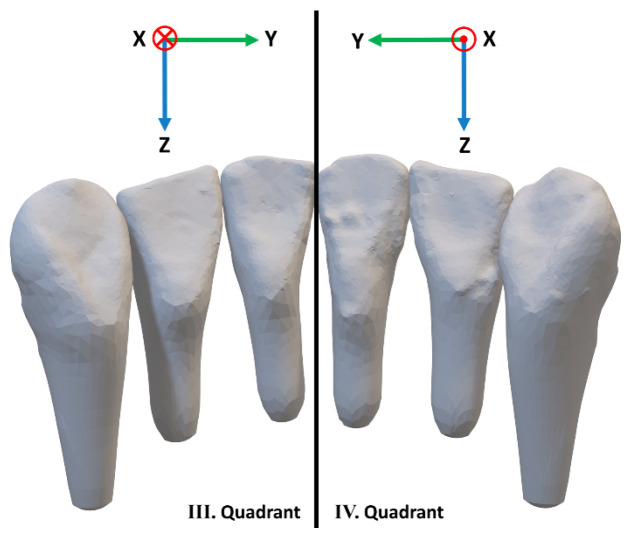
The defined coordinate system for the third and fourth quadrant, viewed from the lingual aspect. In both quadrants, the positive Y-axis indicates a mesial movement and the positive Z-axis indicates an intrusion. In the left quadrant, a positive X-axis indicates a labial movement; conversely, in the right quadrant, a positive X-axis indicates a lingual movement. Due to technical reasons, it was not possible to apply the Tweed convention to this coordinate system.

To analyze the effects of force and torque, a three-dimensional coordinate system was defined for each quadrant (Figure 2). The X-axis lies in the labiolingual plane, the Y-axis in the mesiodistal plane, while the Z-axis lies in the vertical plane. A positive value on the Y-axis indicated a mesial movement towards the midline, while a negative Y-value indicated a movement distally, away from the midline. According to the right-hand rule, in the lower left quadrant (33, 32, 31 according to the Fédération Dentaire Internationale (FDI) notation), a positive X-value indicated a labial movement and a negative X-value indicated a lingual movement. Conversely, when considering the lower right quadrant (43, 42, 41 according to the FDI notation), a positive X-value denoted a lingual movement and a negative X-value denoted a labial movement. In both quadrants, a positive Z-value illustrated an intrusion and a a negative Z-value indicated an extrusion. Thus, moments around the X-axis are indicative of mesiodistal tipping, moments around the Y-axis describe labiolingual torque, while moments around the Z-axis represent rotation around the tooth’s long axis. For a better representation in Figure 2, approximated roots were created with the help of OnyxCeph (OnyxCeph^3TM^, Image Instruments, Chemnitz, Germany). In the actual test setup, the roots were represented by cylinders for a tilt-free guidance in the drill holes of the measurement table.

### 2.3. Statistical Analysis

The data from these measurements were analyzed using Excel 2016 (Microsoft Corporation, Redmond WA, USA), and OriginPro 2022b (OriginLab Corporation, Northhampton MA, USA). Descriptive statistics were performed in IBM SPSS 27 (IBM Corp., Armonk, NY, USA).

## 3. Results

The forces and moments measured during vertical loading at 0.1, 0.2, and 0.3 mm for the respective retainer groups are depicted in Table 2. The values of the individual measurements at the respective positions (0.1 mm, 0.2 mm, and 0.3 mm) were averaged for this purpose. This was carried out separately for each retainer and each tooth. The application of displacement caused forces and moments to arise in all three spatial planes.

**Figure 3:**
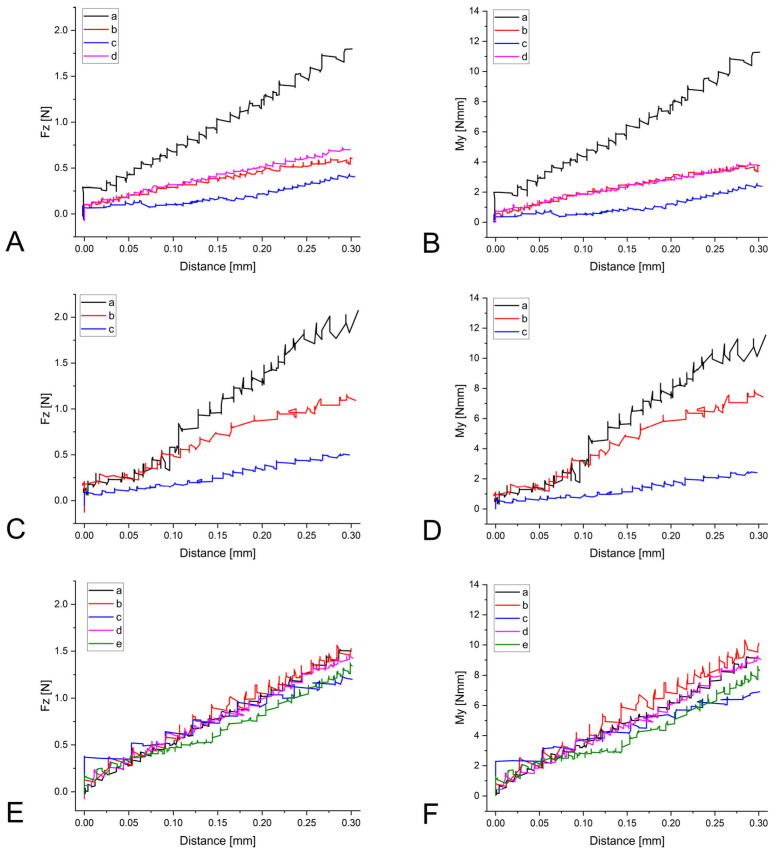
Measured force F_z_ [N] (left) and torque M_y_ [Nmm] (right) for all retainer types R1 (**A**,**B**), R2 (**C**,**D**), and R3 (**E**,**F**), exemplarily shown at the canines. The different measurements (a–e) are distinguished by color. While the curves for R3 (**E**,**F**) appear more homogenous, there are noticeable differences between measurements concerning R1 and R2.

Two aspects are examined in more detail. In the following, the focus is placed on F_z_ and M_y_: the vertical force F_z_ is the direct acting component of the vertical displacement in case of mastication, while ITMs manifest as a labiolingual torquing moment (M_y_). A curve of the measurements of all three analyzed retainers with the canines as reference teeth can be seen in Figure 3. The other samples showed comparable behavior.

### 3.1. Vertical Force

An increase in vertical displacement was accompanied by an increased vertical force F_z_. At all levels of displacement, the lowest F_z_ values were observed in the R1 group with the canines as reference teeth (0.35 ± 0.24 N, 0.61 ± 0.45 N, and 0.88 ± 0.63 N), whereas the highest F_z_ values were found with the lateral incisors as reference teeth in the R2 group for 0.1 mm displacement and in the R3 group for further displacements (1.04 ± 0.71 N, 2.06 ± 1.09 N, and 3.28 ± 1.73 N). The highest interquartile range (IQR) was observed when analyzing the lateral incisors, followed by the canines, whereas the central incisors showed a low IQR.

**Figure 4:**
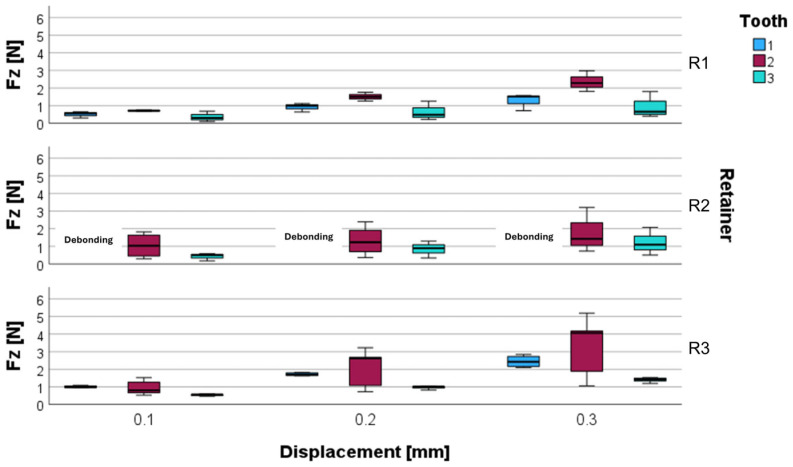
Vertical force F_z_ for different retainer materials (R1, R2, R3) and different reference teeth (central incisor 1, lateral incisor 2, and canine 3) at a displacement of 0.1 mm, 0.2 mm, and 0.3 mm. The force F_z_ was measured at the center of force in N.

Overall, R1 showed the lowest F_z_ values for the central incisors and canines and comparatively low values for the lateral incisors. The results are summarized in Figure 4 and additionally shown in Table 2.

### 3.2. Labiolingual Moment

Labiolingual moments (M_y_) increased in relation to vertical displacement as well. The lowest M_y_ values were observed in the R1 group with the central incisors as reference teeth at all levels of displacement (0.67 ± 0.10 Nmm, 1.43 ± 0.29 Nmm, and 2.01 ± 0.43 Nmm). The highest M_y_ values were found in the R2 group with the lateral incisors as reference teeth at 0.1 mm displacement (4.67 ± 4.79 Nmm), while the highest M_y_ values arose in the R3 group with the lateral incisors as reference teeth (9.27 ± 5.86 Nmm, and 15.91 ± 9.71 Nmm) at 0.2 mm and at 0.3 mm displacement. Similarly to what was observed for the vertical force F_z_, lateral incisors exhibited the highest IQR for M_y_ in almost all groups, followed by the canines, while the central incisors showed a low IQR.

**Figure 5:**
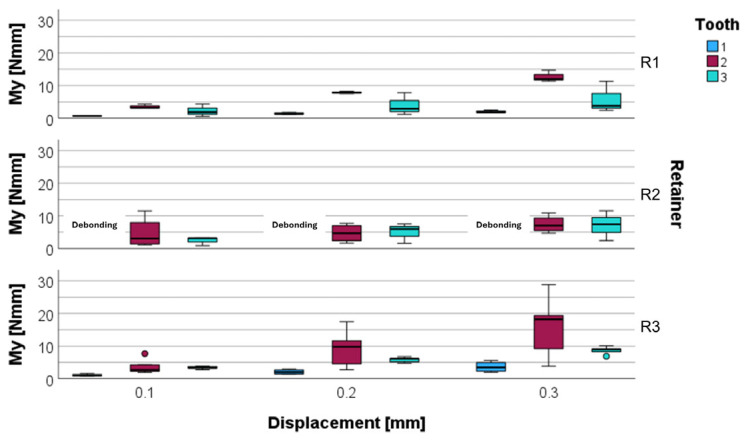
Labiolingual torque M_y_ for different retainer materials (R1, R2, R3) and different reference teeth (central incisor 1, lateral incisor 2, and canine 3) at a displacement of 0.1 mm, 0.2 mm, and 0.3 mm. The moment M_y_ was measured at the center of force in Nmm.

Overall, R1 showed the lowest M_y_ values for the central incisors and canines and comparatively low values for the lateral incisors at an initial displacement. Upon further displacement, R2 showed lower moment development than R1 at the lateral incisors. The results for M_y_ are depicted in Figure 5 and additionally shown in Table 2.

There were differences in force–moment expression when analyzing the data from measurements obtained from different reference teeth. The lateral incisors showed the highest forces and moments overall in both intergroup and intragroup comparisons.

## 4. Discussion

This study investigated the resulting forces and moments upon loading of different retainers, considering differences in the individual teeth.

The R1 retainer showed the lowest forces and moments throughout displacement levels and comparably low forces and moments when considering reference teeth. While low force and moment development is favorable when considering physiological tooth mobility, it is also an expression of low resistance to torque and, consequently, of the possible development of ITMs [31,42]. From a clinical point of view, a lower force development with retainers seems to be advantageous, as these should be of a passive nature in order not to cause active tooth movement in the retention phase [24].

**Figure 6:**
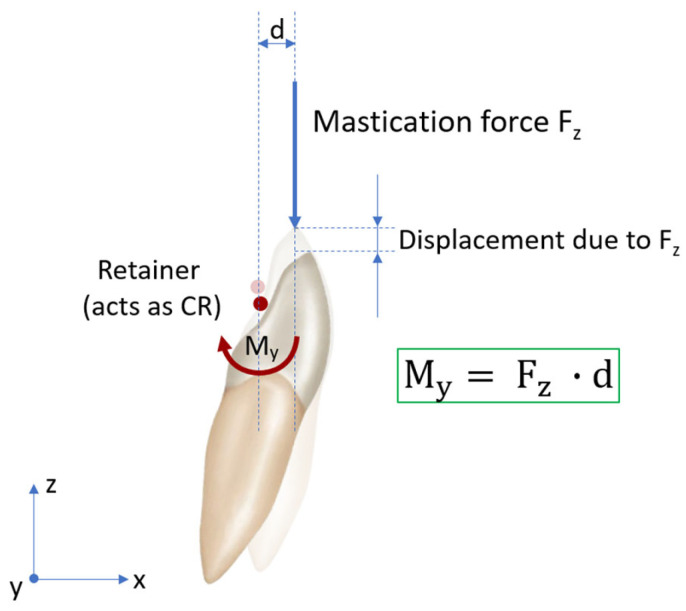
Biomechanical situation of incisal loading during mastication in the presence of a retainer. The retainer serves as center of rotation (CR). The effect is expected to be more pronounced when a twisted retainer wire is used, as the twisted wires are assumed to be less resistant to torsional moments such as M_y_.

The results of the study highlight the direct proportionality of the vertical force component F_z_ and the labiolingual torquing moment M_y_, as well as a general increase in force and moment amplitude with increasing displacement. The original data from the sensor showed a positive M_y_ moment in the third quadrant and a negative M_y_ moment in the fourth quadrant upon extrusion loading. According to the coordinate system (Figure 2), these moments are indicative of a lingual crown torque. Considering an in vivo setting in which mastication forces lead to an intrusion of the mandibular anterior teeth [43], these moments would instead present as a labial crown torque of the affected reference teeth. Both situations reflect the clinical presentation of ITMs, which can manifest as a labial or as a lingual crown torque [17]. The biomechanical situation during masticatory loading on one tooth is shown in Figure 6. The bonding site at which the retainer is fixed is assumed to be the center of rotation (CR). The masticatory force in the vertical F_z_ direction and distance d from the CR create a labiolingual moment M_y_ which might be involved in the development of ITMs.

We noted differences in force–moment development between the different materials being analyzed: lower forces and moments were present in the R1 group for all reference teeth. This is an expression of the low bending as well as torsional stiffness of multistranded retainer wires and of their acceptability with respect to physiological tooth movement [42]. The low stiffness of stranded SS wires has been previously described as a potential risk factor for the development of ITMs [31,44], as it cannot be excluded that a certain amount of torsion is already inherent in the wire due to manufacturing or is acquired while being subjected to masticatory forces [17,18,34]. Multistranded SS and NiTi wires seem to exhibit similar low stiffness, while NiTi exhibits higher flexibility and nonlinear elastic behavior [45]. The higher force and torque values recorded within the R3 group could be because this is the only retainer that did not consist of multiple stranded wires as it was cut out of a single piece of sheet material. In addition, a marked difference could be seen when comparing the force–displacement and moment–displacement curves of the different retainer types. The R3 retainer (Figure 3E,F) produced more homogenous curves throughout all measurements, while the curves varied somewhat between individual measurements when considering R1 and R2 (Figure 3A–D). While the R3 retainer is completely CAD/CAM-manufactured and cut from a single NiTi sheet, the R1 and R2 retainers were bent using pliers and finger pressure, respectively. This could indicate that conventional retainers, being manufactured by humans rather than machines, are inherently less consistent in a testing situation compared to CAD/CAM-manufactured products.

We also noted differences in debonding behavior between different retainer materials and reference teeth. The highest number of debondings occurred in the R2 group, where they also occurred at lower displacements than in groups R1 and R3. This may be due to the flattened rectangular shape of the R2 retainer, which may not allow sufficient adhesion or even form fit between the composite and the wire surface. The more pronounced three-dimensional profile of the other two materials may allow for a better retention of the composite to the metal surface. In addition, the R2 retainer appears to have been rolled as part of its manufacturing process while R1 has not. This signifies that the R1 retainer not only has a better form fit but allows for better adhesion of bonding and composite to its strands. The teeth most frequently affected by debondings were the central incisors, even though these teeth showed the lowest force–moment developments in measurements without debondings.

**Figure 7:**
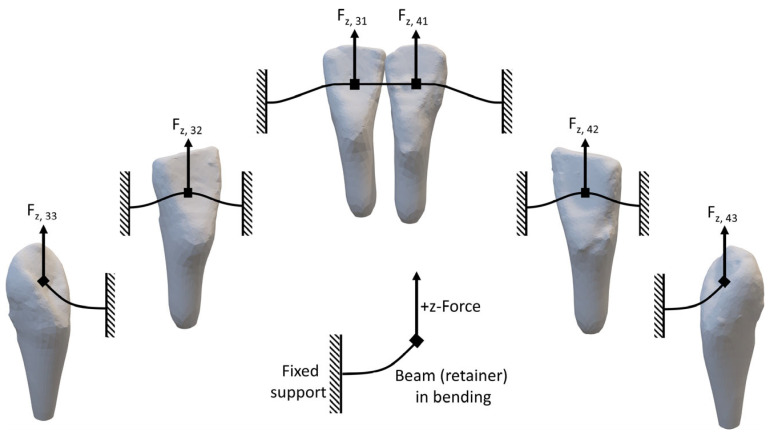
The lower anterior segment during vertical mastication loading causes a force in the negative F_z_ direction. Arrows indicating the vertical force-components on individual teeth (33, 32, 31, 41, 42, 43 according to the FDI notation). It becomes evident that the loaded canines (33 and 43) are only supported on one side whereas the lateral incisors (32 and 42) have support from the central incisors as well as from the canines. If the load is applied to the central incisors (31 and 41), the biomechanical support from the neighboring teeth is in between the two previously mentioned cases.

When considering differences between reference teeth, the lateral incisors showed higher forces and moments than the other teeth. While other studies suggest that the different behavior of the lateral incisors could be due to differences in root morphology [46], this influence could be excluded in our study since the roots were shaped uniformly and fixed mechanically into the base plate. It can be assumed that this may be due to the position of the lateral incisors within the retainer and its influence on the dissipation of bearing forces (Figure 7). While mastication forces are being applied to the lateral incisors (teeth 42 and 32), they are mechanically connected by short wire lengths at both sides of the point of force application. This leads to the development of moments, which potentially results in a rotation of the teeth around the retainer. In contrast, when a vertical force is applied to both central incisors (teeth 41 and 31), they are connected to each other by a flexible section of free wire mesially, with the mechanical fixation to the frame featuring only on their respective distal side. This leads to lower stress in the retainer and thus to lower amounts of generated moments. While the central and lateral incisors are supported on both sides by the retainer, the canines are only supported on one side. The canines are therefore less restricted in their movement compared to the incisors, leading to a lower force development F_z_ at the canines, which in return means that the retainer effect decreases. Additionally, the distance of free wire between the points of force application is longer between the canines than in all other setups, leading to higher moment developments M_y_ due to the longer lever arms.

Several clinical studies have been performed to examine patient-dependent features of ITMs [18,26], while biomechanical in vitro studies analyzed the behavior of retainer materials under mechanical stress or loading. Sifakakis et al. [35] analyzed labiolingual and intrusive forces acting on three different stainless-steel retainer wires. Similarly to our results, they found that displacements of 0.2 mm in the vertical plane caused forces of 1 N or larger to arise. These forces would be sufficient to produce tooth movements despite the presence of a bonded retainer. In a follow up study [34], the same research group analyzed the effects of vertical force loading and unloading on different retainer materials to simulate the effects of mastication. All retainer types showed residual forces and moments after the loading phase, meaning that they had become active and that, consequently, ITMs could occur in a clinical setting. Additionally, it was hypothesized that the twist direction of the wire strands may have an influence on torque resistance based on the assumption that it is significantly more difficult to strand a wire further than to untwist it against the direction of stranding. This effect could cause a different torsional moment on the teeth between the left and right lower quadrant and, in turn, a more pronounced ITM manifestation on the twisted side of the retainer. This could be a decisive factor as to why some patients only develop ITM on one side, although further studies are needed to scientifically confirm this theory. Seide et al. [30] analyzed lingual retainers by applying horizontal force in vitro. They hypothesized that ITMs could occur due to unwinding of the wire at an intact wire–composite interface or due to rotation of the tooth around the wire at a compromised wire–composite interface. This seems like a plausible explanation for this phenomenon. In contrast to the aforementioned study [30], we chose to apply a vertical force, since vertical forces (e.g., due to mastication) seem to act more directly on the retainer itself rather than horizontal ones. Our data seems to confirm a direct relationship between an increase in vertical force F_z_ and labiolingual torquing moment M_y_, which is how ITMs present clinically. The application of a vertical displacement caused forces and moments to arise in all spatial planes in accordance with the findings of Cooke and Sherriff [32]. This indicates that the FRANS offers a good simulation of the in vivo situation, since a bonded retainer wire is subjected to multiple forces and moments, from multiple directions, such as vertical forces due to mastication and horizontal forces due to tongue function.

It is important to allow for a certain degree of horizontal and vertical tooth mobility during permanent retention, since failure to do so would result in detrimental effects, such as inactivity-related atrophy of the PDL [47]. This was already recognized by Schwarze et al. [33] in 1995, who performed in vitro tests on various retainer materials to verify their compatibility with physiological tooth movements. They found the 0.0155” TwistFlex wire (Dentaflex, Dentaurum, Ispringen, Germany) to be acceptable in both an in vitro and an in vivo setting, even though tooth mobility was limited by the retainer. More recently, Roser et al. [42] compared the restriction of physiological tooth mobility in the presence of different retainer materials, including those used in our study in groups R1 and R3, in an in vitro investigation. They found that the NiTi retainer allowed more tooth mobility than the SS retainer but deemed both materials acceptable in regard to physiological tooth mobility.

The same research group also compared conventional SS retainers (analogous to group R1) to various CAD/CAM retainers (including those analyzed in group R3) in respect to simulated aging and maximum load capacity [40]. In contrast to their findings, we found the NiTi retainer group to have the lowest bond failure rate of those analyzed. This may be due to the use of simulating aging in the study by Roser et al. [40] and to differences in bonding protocol between studies: in our study, the surface of the specimen was not preconditioned, as synthetic resin (Grey V4) was used, and a sufficient bond strength with orthodontic resins was implied due to the characteristics of both materials. Vertical force values of 3.31 N and labiolingual torque values of up to 15.91 Nmm were reached, and debonding of the wire did not occur. This lies within the range of regular adhesive bond strength [48].

In the FRANS setup, the measurement table can be moved in the sagittal dimension. In a research setting, this allows for simulations of forces and moments acting upon protruded or retroclined lower incisors or even for a comparison of buccal and lingual mechanics. The FRANS also allows for a continuous application of displacement, and, consequently, of forces and moments, as opposed to an incremental application. This leads to a more accurate simulation of in vivo conditions, since biting forces are not exerted in constant increments in nature.

A limitation of in vitro setups in general, including the FRANS, is that the conditions are per definition different to those in vivo: the influence of saliva, pH variation, and repeated masticational or parafunctional forces cannot be replicated exactly. In our study, we did not consider an additional embedding of the teeth in a PDL-like structure. For this reason, the resistance of the moving teeth can therefore only be seen as partially representative of the clinical situation. While displacements of up to 0.2 mm have already been reported in in vivo studies on maxillary central incisors [49], as well as in in vitro biomechanical studies [50], our specimens were displaced further, reaching 0.3 mm, to compensate for the missing PDL. We did not use human teeth in our measurements, instead using teeth fabricated from 3D-printed resin. This leads to a more homogenous specimen group, since individual structural and anatomical differences can be excluded by 3D-printing the same model for all specimens. However, this also implies differences to the in vivo situation since the bond strength between the bonding composite and the 3D-printed resin will inevitably be different to that to the preconditioned enamel surface.

In comparison to the other previously described [31,34,35] biomechanical analysis systems, the FRANS offers the possibility to exert displacement only in one dimension. Applying displacement in multiple spatial planes is crucial if dynamic measurements need to be performed, although the absence of unwanted movement in other dimensional planes may be an important advantage in other more static measurement setups. A further limitation is that the FRANS only allows for the analysis of two single, biomechanically isolated reference teeth at a time. While this provides important insight into the loading behavior of each tooth of the mandibular anterior arch, it is not possible to analyze all teeth at the same time or to compare different reference teeth directly because of the design of the FRANS. A comparison between different reference teeth (e.g., canines vs lateral incisors) can only be undertaken indirectly by comparing separate measurements.

Taking into account the limitations of the setup, the following observations might be of clinical relevance. CAD-CAM-manufactured retainers showed more consistent force responses, suggesting better predictability during loading. Predictability may be an important factor for clinicians considering which retainer material to use. This study noted higher debonding rates for the R2 group, likely due to its flattened shape, which affects adhesion. The lateral incisors showed the highest development of forces and moments: these may propagate along the retainer and cause debondings or inadvertent movement of other teeth as well. Furthermore, it is important to ensure that these teeth are not subject to premature occlusal contacts to exclude even higher levels of forces arising due to masticational or parafuncional loading.

While there have been no clinical reports of ITMs with retainers other than multistranded SS retainers, to the authors’ knowledge, the forces and moments recorded in this study could be sufficient to produce unwanted tooth movements with all analyzed retainer materials, especially when considering displacements of 0.2 mm or larger. Clinicians must be mindful of this implication when examining patients during the retention phase.

Further research is needed to exclude the possibility of ITMs arising from materials other than SS and to test the biomechanical properties of novel CAD/CAM-developed retainer materials. While this present experimental setup simulated the effects of vertical displacement on two pairs of teeth at a time, future research could analyze the effects of a vertical displacement acting uniformly on the entire lower anterior segment.

## 5. Conclusions

The FRANS is a measurement system that can be reliably used to research the biomechanical properties of retainer wires and other orthodontic materials. In this present study, the development of forces and moments caused by a vertical displacement on different reference teeth was analyzed.

The forces and moments are expressed differently on each tooth in the lower anterior segment, with the lateral incisors in particular being exposed to higher force and torque levels than their neighboring teeth.

Our results show that a certain degree of labiolingual tooth movement can be expected when applying vertical forces even though bonded retainers are present. While R1 developed the lowest forces and moments throughout, all analyzed retainer materials showed a force and moment development sufficient to lead to the expression of unwanted torque movements such as ITMs. Clinicians should be mindful of this implication and check for ITM-like movements associated with retainers of materials other than stainless-steel. The clinician might also keep in mind that different retainer materials show different levels of force and moment development and that these characteristics can impact physiologic tooth mobility and PDL health.

## Figures and Tables

**Table 1:** Retainer characteristics of the retainer wires analyzed in this study. The material, cross-sectional dimensions, shape, and manufacturing process are listed.

Retainer	Material	Dimension	Shape	Manufacturing Process	Included Measurements
R1	SS	0.0215″	stranded, round	bent with pliers	n _(R1)_ = 10 n _(R1, central incisors)_ = 3 n _(R1, lateral incisors)_ = 3 n _(R1, canines)_ = 4
R2	SS	0.010″ × 0.029″	stranded, rectangular, flat	adapted by hand	n _(R2)_ = 7 n _(R2, central incisors)_ = 0 n _(R2, lateral incisors)_ = 4 n _(R2, canines)_ = 3
R3	NiTi	0.014″ × 0.014″	rectangular	CAD/CAM laser cut from sheet	n _(R3)_ = 14 n _(R3, central incisors)_ = 4 n _(R3, lateral incisors)_ = 5 n _(R3, canines)_ = 5

SS: Stainless-steel; NiTi: nickel–titanium; CAD: computer-aided design; CAM: computer-aided manufacturing.

**Table 2:** Calculated mean and standard deviation for F_z_ [N] and M_y_ [Nmm] measured at the center of force (CoF) for different displacements (0.1 mm, 0.2 mm, 0.3 mm), different retainers (R1, R2, R3) and different reference teeth (1, 2, 3). Data showing mean over left and right quadrant.

Displacement [mm]	Retainer	Tooth	F_z_ [N]	M_y_ [Nmm]
			Mean (SD)	Mean (SD)
0.1	R1	1	0.50 (0.18)	0.67 (0.10)
		2	0.71 (0.04)	3.52 (0.70)
		3	0.35 (0.24)	2.16 (1.59)
	R2	2	1.04 (0.71)	4.67 (4.79)
		3	0.42 (0.22)	2.41 (1.34)
	R3	1	1.01 (0.06)	1.10 (0.34)
		2	0.96 (0.42)	3.80 (2.36)
		3	0.55 (0.05)	3.41 (0.42)
0.2	R1	1	0.92 (0.25)	1.43 (0.29)
		2	1.50 (0.25)	7.86 (0.34)
		3	0.61 (0.45)	3.70 (2.85)
	R2	2	1.30 (0.85)	4.67 (2.80)
		3	0.84 (0.48)	5.02 (3.09)
	R3	1	1.72 (0.09)	2.10 (0.76)
		2	2.06 (1.09)	9.27 (5.86)
		3	0.97 (0.09)	5.82 (0.82)
0.3	R1	1	1.27 (0.48)	2.01 (0.43)
		2	2.35 (0.59)	12.68 (1.75)
		3	0.88 (0.63)	5.30 (4.04)
	R2	2	1.69 (1.06)	7.42 (2.65)
		3	1.22 (0.80)	7.13 (4.57)
	R3	1	2.45 (0.34)	3.66 (1.63)
		2	3.28 (1.73)	15.91 (9.71)
		3	1.40 (0.13)	8.70 (1.19)

## Data Availability

The raw data supporting the conclusions of this article will be made available by the authors on request.
